# Co-creation of a toolkit to assist risk communication and clinical decision-making in severe preeclampsia: SPOT-Impact study design

**DOI:** 10.1080/16549716.2024.2336314

**Published:** 2024-05-08

**Authors:** Hannah Brown Amoakoh, Bregje C. De Kok, Linda Lucy Yevoo, Klaartje M. Olde Loohuis, Emmanuel K. Srofenyoh, Daniel K. Arhinful, Koiwah Koi-Larbi, Kwame Adu-Bonsaffoh, Mary Amoakoh-Coleman, Joyce L. Browne

**Affiliations:** a Department of Epidemiology, Noguchi Memorial Institute for Medical Research, University of Ghana, Accra, Ghana; b Department of Global Health and Bioethics, Julius Center for Health Sciences and Primary Care, University Medical Center Utrecht, Utrecht, Netherlands; c Anthropology Department, University of Amsterdam, Amsterdam, Netherlands; d Department of Obstetrics and Gynaecology, Greater Accra Regional Hospital, Accra, Ghana; e Executive Council, Action on Preeclampsia Ghana, Accra, Ghana; f Department of Obstetrics and Gynaecology, University of Ghana Medical School, Accra, Ghana

**Keywords:** Respectful maternal care, patient-centered care, shared-decision making, low-resource setting, hypertensive disorders of pregnancy

## Abstract

Globally, the incidence of hypertensive disorders of pregnancy, especially preeclampsia, remains high, particularly in low- and middle-income countries. The burden of adverse maternal and perinatal outcomes is particularly high for women who develop a hypertensive disorder remote from term (<34 weeks). In parallel, many women have a suboptimal experience of care. To improve the quality of care in terms of provision and experience, there is a need to support the communication of risks and making of treatment decision in ways that promote respectful maternity care. Our study objective is to co-create a tool(kit) to support clinical decision-making, communication of risks and shared decision-making in preeclampsia with relevant stakeholders, incorporating respectful maternity care, justice, and equity principles. This qualitative study detailing the exploratory phase of co-creation takes place over 17 months (Nov 2021-March 2024) in the Greater Accra and Eastern Regions of Ghana. Informed by ethnographic observations of care interactions, in-depth interviews and focus group and group discussions, the tool(kit) will be developed with survivors and women with hypertensive disorders of pregnancy and their families, health professionals, policy makers, and researchers. The tool(kit) will consist of three components: quantitative predicted risk (based on external validated risk models or absolute risk of adverse outcomes), risk communication, and shared decision-making support. We expect to co-create a user-friendly tool(kit) to improve the quality of care for women with preeclampsia remote from term which will contribute to better maternal and perinatal health outcomes as well as better maternity care experience for women in Ghana.

## Paper Context

- Adverse maternal and perinatal outcomes is high for women who develop preeclampsia remote from term (<34 weeks). To improve the quality of provision and experience of care, there is a need to support communication of risks and treatment decisions that promotes respectful maternity care.
- This article describes the methodology deployed to cocreate a user-friendly tool(kit) to support risk communication and shared decision-making in the context of severe preeclampsia in a low resource setting.

## Background

Global and local efforts and interventions have resulted in substantially improved access to maternal healthcare, including increased antenatal care coverage and facility-based deliveries [1]. This has contributed to reduced maternal and perinatal death rates, particularly after the introduction of the Millennium Development Goals (MDGs) [2]. The MDG 4 sought to reduce the under-five mortality rate by two-thirds and MDG 5 to reduce maternal mortality ratio by three-quarters. Available estimates suggest that globally, maternal deaths reduced by 34% between 2000 and 2020 [3]. Still, maternal mortality remains unacceptably high at 223 per 100, 000 live births [3]. An estimated 99% of the maternal, and perinatal deaths occur in low- and middle-income countries (LMICs) [4,5]. The 2020 annual average MMR among LMICs was 232 deaths per 100,000 live births [6]. Indeed, many LMICs, including Ghana, are behind in achieving the Sustainable Development Goal (SDG) to reduce maternal deaths to less than 70 per 100,000 live births by 2030. Furthermore, neonatal deaths represent a growing proportion of all under-five deaths [7].

**Figure 1.**
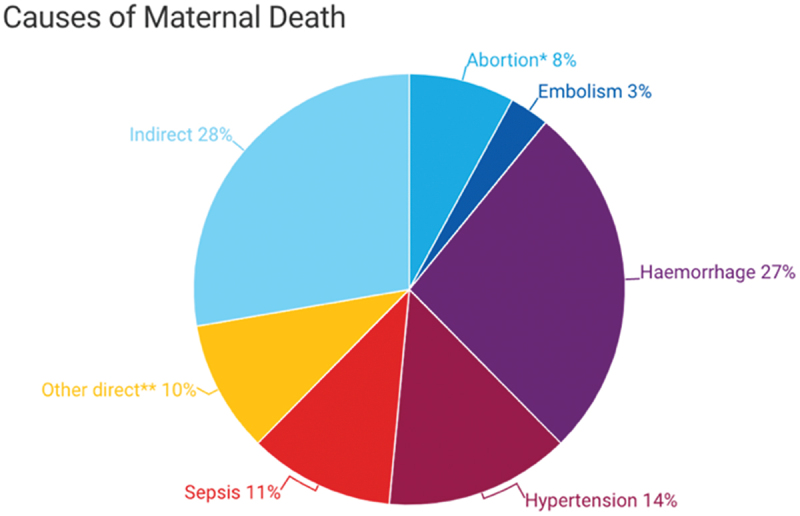
Direct obstetric cause of maternal mortality.

*Source: UNICEF Data 2023* [3]

Globally, most maternal deaths are due to direct obstetrics causes (see Figure 1), which include hemorrhage, abortion, hypertensive disorders of pregnancy, sepsis, ectopic gestation, and embolism [8].

### Hypertensive disorders of pregnancy (HDP)

Most maternal deaths are preventable. An estimated 14% of maternal deaths are due to HDP [9]. HDP are a group of high blood pressure-related disorders that occur during pregnancy. There are several types of HDP with varying degrees of complications [10] (see Table 1), with risk of adverse maternal or neonatal outcomes increasing when occurring remote from term (<34 weeks). Maternal adverse outcomes include intensive care unit (ICU) admission and maternal mortality. In addition, long-term outcomes associated with HDP include an elevated risk of repeat HDP in future pregnancies and high incidences of non-communicable diseases like cardiovascular disease in an affected woman and her offspring [11]. HDP is responsible for 70,000 maternal deaths globally each year [12] and is also associated with severe adverse perinatal outcomes [4], including approximately 500,000 fetal and neonatal deaths. Adverse perinatal outcomes include prematurity, small-for-gestational age, infection, and neonatal ICU admission [4,13–17].

**Table 1.** The various types of hypertensive disorders of pregnancy (HDP).

Type of HDP	Time of diagnosis	Features
Chronic hypertension	<20-week gestation	Elevated blood pressure: systolic/diastolic >140/90 mmHg
Gestational hypertension	>20-week gestation	Elevated blood pressure: systolic/diastolic >140/90 mmHg
Preeclampsia	>20-week gestation	Elevated blood pressure with*: proteinuria and/or maternal acute kidney injury, liver dysfunction, neurological features, hemolysis, or thrombocytopenia with/or without HELLP syndrome, or fetal growth restriction
Eclampsia	>20-week gestation	Same as preeclampsia, with the addition of fits

*Current ISSHP definition also allows for the diagnosis of preeclampsia in the absence of hypertension, but with one of the organ damage symptoms.

*Source: ISSHP, 2018* [10].

Ghana, a lower middle-income country, has seen remarkable declines in maternal deaths over the years [18], but her maternal mortality ratio is still high [19], with currently available estimates of 263 maternal deaths per 100,000 live births [20]. HDP is one of the leading causes of maternal and perinatal deaths in Ghana. A 2014 report noted that 30% of maternal deaths in Ghana were due to HDP [16,21], and another study conducted in the Great Accra Region identified that 14.6% of maternal mortality cases are caused by (pre)eclampsia [22]. Another study conducted in Kumasi showed that 87% of women admitted to a tertiary facility with HDP experienced adverse maternal or perinatal outcomes including maternal mortality, prolonged maternal hospital stays, low Apgar score and birth weight, stillbirths, and neonatal ICU admissions [4].

### Management of HDP

Management of HDP includes treatment with antihypertensive medicines, monitoring of maternal and fetal condition and timely delivery [12,23], as the only way in which preeclampsia can be cured is through delivery. There is general agreement that all patients with severe preeclampsia should be delivered if the disease develops after 34 weeks of gestation or if there is evidence of maternal or fetal distress [24]. Severe early-onset preeclampsia before 26 weeks is associated with high maternal morbidity and very poor perinatal outcomes, even in high-income settings [25]. This leaves the gestational age range of 26 to 34 weeks as key ‘grey zone’ in obstetric care that needs to be elucidated. Several guidelines recommend that pregnant women with a mild form of preeclampsia should deliver at 37 weeks to ensure better maternal outcomes [26]. However, a Danish study indicated that the pregnancy should be carried to term whenever possible [27]. In this decision, health providers must weigh the risks of premature birth for the baby and benefits of prolonging pregnancy to allow further growth, against the impact of worsening of preeclampsia for maternal and perinatal health [26].

Good clinical management of women with HDP is thus essential, but complex. Evidence suggests that the quality of maternity care is often suboptimal in LMICs, including Ghana, because of persistent material and human resource shortages [28]. As such, ‘risk-based care’ is increasingly being employed to address the resource problem and improve outcomes, as reflected by a rapidly growing field of prediction research, facilitated by technological developments in information sciences and increased availability of (big) data [29–32]. In this approach, an individual’s predicted risk of an (adverse) outcome allows health care workers to triage patients into low, moderate, or high risk, with corresponding options for intervention to prevent or treat disease. Thus, risk prediction can facilitate providing quality health care for the right person at the right moment, which is crucial especially in resource-constrained settings. Regrettably, there is limited research in LMICs including Ghana on *how* risks based on prediction models can assist maternal and newborn healthcare professionals to provide better quality of care and improve maternal and perinatal health outcomes and care experiences for women with HDP.

## Quality of care

**Figure 2.**
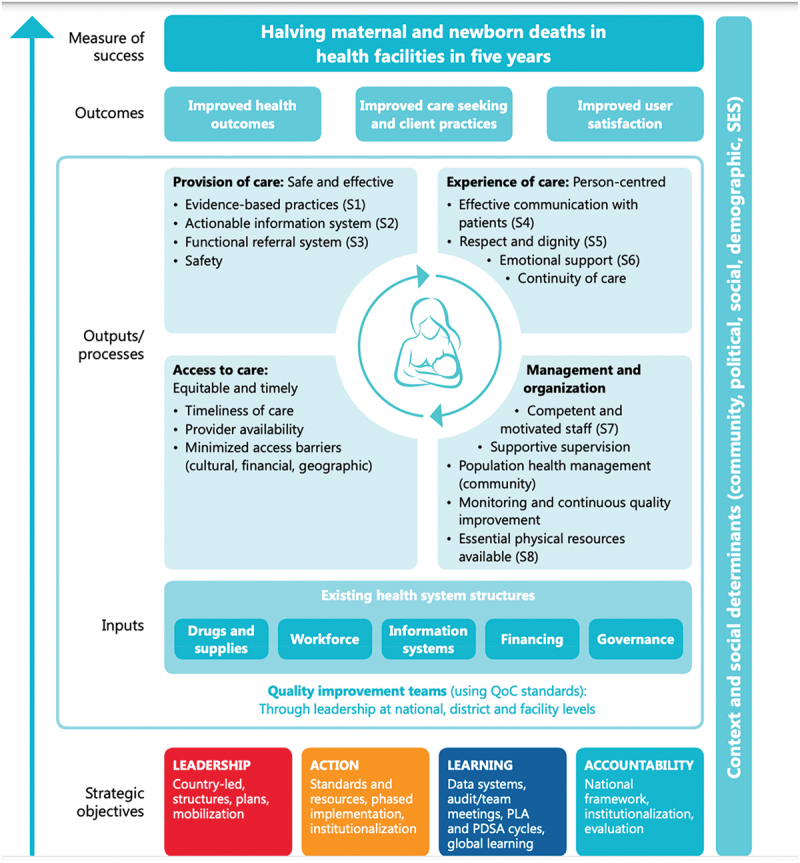
WHO framework for improving quality of maternal and newborn care in health facilities.

*Source: WHO 2016* [36]

Following the WHO (World Health Organization) *Standards for improving maternal and newborn care in health facilities* [33], quality of care is defined as ”the degree to which health services for individuals and populations increase the likelihood of desired health outcomes”. This means that quality care is safe, effective, timely, efficient, equitable and people-centered, meaning care that considers the preferences and aspirations of clients and the culture of their community [34]. These WHO Standards include a quality-of-care framework that consists of eight domains of care, pertaining to provision and experience of care (see Figure 2).

According to the recent WHO quality of antenatal and intrapartum care frameworks [35], determinants of positive birth experiences and respectful quality care include cultural sensitivity, emotional support for women and their families, and effective communication which includes clear explanations regarding interventions or outcomes, informed choices, and involvement in decision-making. Underlying this attention to positive birth experience and respect is the rapidly growing body of literature that documents that disrespectful care practices are widespread globally, and particularly in LMICs [36]. However, this recent paradigm shift that makes patient-centered, respectful care and positive birth experiences central to quality care requires guidance regarding how to apply these principles.

People or (patient)-centered care means ensuring that health services are tailored to people’s needs and are provided in partnership with them rather than simply given to them; it means care where people, families, and communities are respected, informed, engaged, supported, and treated with dignity and compassion [37,38]. Shared decision-making (SDM) has been proposed to be an integral part of patient-centered care. SDM is a collaborative process, ‘by which clinicians and patients (and/or their carers and families) come to a clinical decision regarding the next step to take in a patient’s health care’ [39]. SDM involves a two-way exchange which brings together: *i*. medical evidence and the clinician’s expertise on treatment options, risks, and benefits and *ii*. what the patient knows best: their preferences, personal circumstances, goals, values, and beliefs [39]. Effective risk communication has been identified as key precursor to shared decision-making [40].

However, it is not yet well known *how* to engage in people-centered care and SDM or effective risk communication, in LMICs. There are few detailed observational studies of shared decision-making practices or risk communication in LMICs, including Ghana; existing studies tend to rely on interviews or surveys (for exceptions see [41,42]). This is an important research gap, because shared decision-making may not be universally acceptable or appropriate due to for example providers’ and clients’ expectations, habits, or the health system context. The WHO (2018) recognizes that women in low-income countries are less likely to demand involvement in decision-making over their childbirth [43]. Cultural sensitivity and contextualization are required when exploring and implementing SDM in (clinical) practice. In certain contexts, and situations, it could be more suitable to incorporate only *some* elements of SDM or the related broader concept of patient-centered care.

Several barriers complicate SDM in LMICs [44,45]. These include the highly hierarchical societal and health systems context marked by large socio-economic and power differences between providers and patients, relatively low education levels, and shortage of resources including time necessary for in-depth conversations [46,47]. Furthermore, SDM in maternal health is complicated by the profound psychological and social meanings of pregnancy and pregnancy loss, which may greatly affect women and their relationships with partners, relatives, and community members [48]. Medical *and* social risks need to be considered and it may not be up to the woman alone to take decisions regarding management of her pregnancy such as premature termination of pregnancy. Such termination may be associated with abortion which is highly stigmatized in many LMICs, where cesarean sections also carry stigma [49].

A final layer of complication related to SDM concerns risk communication. Risk communication is never straightforward due to the general difficulty of interpreting clinical risk statistics, the inherent uncertainties in predicting future events, and the difficulty to translate predicted risk based on group averages to the individual risk a woman faces, as documented in research about lay interpretations of risks [50].

Whilst it is clear that SDM and risk communication are complex matters, there is a paucity of knowledge on how maternal and newborn healthcare providers and clients in low resources settings like Ghana can engage in SDM, and how healthcare providers currently communicate risk(s) and treatment decisions to pregnant women with HDP and their family members. We sought to address these knowledge gaps through the Severe Preeclampsia Adverse Outcome Triage (SPOT) – Impact study [51].

## Study aim and objectives

The overarching study aim was to develop a simple, user-friendly, and low-cost tool(kit) based on risk estimation that can be easily used in an LMIC hospital setting using a co-creation approach.

The SPOT tool(kit) will go beyond a simple risk prediction algorithm and will be designed to facilitate a (shared) decision-making process aligned to WHO’s quality of care framework. In this way, the predicted risk of adverse maternal and perinatal health outcomes for a woman becomes a starting point for a conversation between providers and their clients. The estimated risks of adverse outcomes will be obtained from another arm of the SPOT study that seeks to validate the full and mini-preeclampsia integrated estimate of risk score (PIERS) prediction models for women with HDP [51]. The SPOT tool(toolkit): using the term tool, we refer to a product that incorporates (absolute) risk prediction, communication, and shared decision-making; by toolkit, we refer to a range of products that will include tools for training and implementation and possible scale-up of the tool.

The specific study objectives pertained to several knowledge gaps that needed to be filled before we could develop the SPOT-tool(kit) and are as follows:

1. To assess barriers to, and facilitators of, meaningful participation in co-creation.
2. To assess understandings, preferences, and suitable forms of a) risk communication and b) shared decision-making (SDM).
3. To assess personal, interpersonal and system barriers to, and facilitators of, SDM.

Specific research questions that address these objectives, are listed in Table 2.

**Table 2.** Overview of knowledge gaps, methodological approach, and relevant stakeholders participating.

Knowledge gaps to be addressed: research objectives and questions	Methods/tools	Stakeholders involved
1. Assess barriers to and facilitators of meaningful participation in co-creation. 1.1. How might stakeholders’ knowledge or skills gaps; motivation; relationships, trust and power-dynamics become barriers to meaningful collaboration and co-creation? 1.2. How can we address identified barriers to, and strengthen facilitators of, co-creation and involve all stakeholders in a meaningful way?	● Literature review ● FGDs, including participatory methods: problem trees; ● Semi-structured interviews ● Observations during a) APEC webinars and b) co-creation activities	● Health providers (obstetricians; midwives) ● Facility managers ● Survivors ● Male partners ● Relatives
2. Assess understandings, preferences and suitable forms of a) risk communication and b) shared decision-making 2.1 How are HDP associated risks currently communicated by professionals and responded to by clients, spouses, or relatives during consultations? 2.2. What are providers’ & women’s preferences regarding risk communication related to HDP? 2.3. How are treatment-related decisions currently communicated by professionals and responded to by clients, spouses, or relatives during consultations? 2.4. What do different stakeholders understand by, and associate with, the term SDM? 2.5. Are alternative terms more appropriate (e.g. collaboration; patient-centered care; effective communication)? 2.6. What are providers’ & women’s preferences regarding decision-making concerning the treatment of women with HDP? 2.7. What modes of involving women in clinical decision- making do health providers and women consider appropriate and relevant? 2.8. What, if any, might be the unintended consequences of shared decision-making between providers and women?	● Literature review ● FGDs ● Semi-structured interviews ● Ethnographic observations of care interactions ● Structured conversations ● Conversation analysis: recordings of consultations with women with HDP (pre and post labor, not during labor) ● Embedding of questions in and observations of APECGH webinars.	● Health providers (obstetricians; midwives) ● Health managers ● Survivors ● Women with HDP ● Male partners ● Relatives ● Policy makers
3. Assess personal, interpersonal and system barriers to, and facilitators of SDM. 3.2. What barriers may affect SDM? 3.1. How might these barriers affect SDM? 3.3. How might knowledge or skill gaps, relational aspects (power-dynamics, trust between different stakeholders) or health system factors constitute barriers or facilitators to shared decision-making? 3.4. What, if any, are available guidelines regarding SDM or effective communication, according to protocols and training manuals in Ghana? 3.5. To what extent do providers know of guidelines and have been trained in, regarding SDM and effective communication? 3.6. How can we address barriers to SDM? 3.7. How can we strengthen facilitators of SDM or other forms of patient centred care deemed appropriate to the local context?	● FGDs ● Semi-structured interviews ● Observations during a) APEC-Ghana webinars and b) co-creation FGDs/activities ● Structured conversations ● Ethnographic observations of care interactions ● Conversation analysis: recordings of consultations with women with HDP (pre and post labor, not during labor) ● Document review (for 3.4, 3.5).	● Health providers (obstetricians; midwives) ● Facility managers ● Survivors ● Women with HDP ● Male partners ● Relatives

## Co-creation approach

To develop the SPOT tool(kit), we adopted a co-creation approach: a collaborative approach to development of health services and interventions in which academics work alongside other stakeholders [52]. Co-creation is considered a particularly promising strategy in implementation and health research which could enhance quality of services, adherence to and effectiveness of interventions, and patient experiences [52]. A likely reason for these positive effects is that co-creation enables departure from a ‘one size fits all approach’ [52]. Co-creation empowers users to help tailor interventions to their circumstances and shift from a ‘product-centered’ to ‘experience-centered’ approach [52,53]. Finally, co-creation is thought to produce a sustainable impact due to its emphasis on stakeholder engagement, intersectoral collaboration, power sharing, and ongoing conflict resolution [52].

Key features of co-creation apart from its collaborative knowledge production include a flexible and iterative rather than linear process, in which reflections on the nature of the problem and associated questions continuously inform activities. One challenge is that due to the diversity of possibly competing perspectives that are brought together, co-creation is invariably ‘power-charged and conflict-ridden’ [54]. A review by Greenhalgh et al. (2016) identified three success principles underpinning co-creation [54] that have been built into our co-creation design. These principles are:

(1) *Adopt a systems perspective: Assume emergence, local adaptation, nonlinearity*.
  - *Create room for adaptations, unexpected processes & outcomes.*
  - *Regular process and outcome evaluation; feedback loops, reverting to earlier stages, but balance with time constraints.*
(2) *Consider research as a creative enterprise with human experience at its core*.
  - *Identify methodologies which enable participants to share experiences.*
  - *Find ways to ensure that tool(s) produced reflects a variety of experiences & perspectives.*
(3) *Emphasize process as well as outcomes*.
  - *Invest time and resources in process development to enhance quality and productivity of interactions during co-creation process.*
  - *Pay attention to governance and facilitation arrangements, including leadership style; the nature of relationships, interactions, and dialogue; management of conflict & power dynamics.*

Co-creation can take various forms, partly because it has roots in multiple disciplines and earlier approaches [54], including participatory action research (PAR) [54] and experience-based co-design (EBCD) [55], two traditions that informed our approach. For instance, we incorporated PAR’s focus on power sharing, mutual learning, and empowerment. Experienced-based co-design (EBCD) acknowledges that quality improvement requires that tools, services, and care pathways are designed around the experiences of patients and care providers, so that they appeal and work ‘on a cognitive and emotional level’. EBCD seeks to shape and design objective tools, care pathways and environments as well as subjective experiences by collecting and sharing health workers’ and clients’ experiences through qualitative methods [55].

We will co-create an effective and acceptable tool(kit) that improves care for pregnant women with HDP, based on risk estimations, principles of respectful and patient-centered care (i.e. incorporating women’s preferences, needs and aspirations and the culture of their community) and shared decision-making [35]). The tool would be used by clinicians, and possibly by pregnant women, when risk assessment and clinical decision-making needs to take place. The tool will be co-created with all relevant stakeholders to ensure that it reflects different stakeholders’ needs and experiences and that all relevant stakeholders are on board, including more marginalized groups of women (e.g. women with disabilities).

Design of the co-creation process was informed by the PRODUCES framework [52] to specify the Problem we seek to address; the tool(kit)’s Objective; co-creation Design; end Users; Co-creators; Evaluation approach; and Scalability.

Furthermore, we identified three ethical and quality of care frameworks to guide the co-creation process. First, the tool(kit) should respect the right to health and be acceptable, accessible, available and promote quality of care in terms of clinical best practice and women’s experiences. Second, the tool(kit) should be aligned with respectful maternity care principles and, from a global health justice perspective [56], be inclusive of marginalized populations and reduce or at least not enhance health disparities. The Justice for Global Health Framework recommends four features that signal a more equitable study design:

- Selection of populations (e.g. choose disadvantaged or marginalized populations).
- Equity-oriented question through inclusive process.
- Capacity development at individual and institutional levels.
- Lasting change to reduce health disparities.

Third, the development of the tool(kit) is guided by the WHO quality of care framework for maternal and newborn care [26], as discussed above.

**Figure 3.**
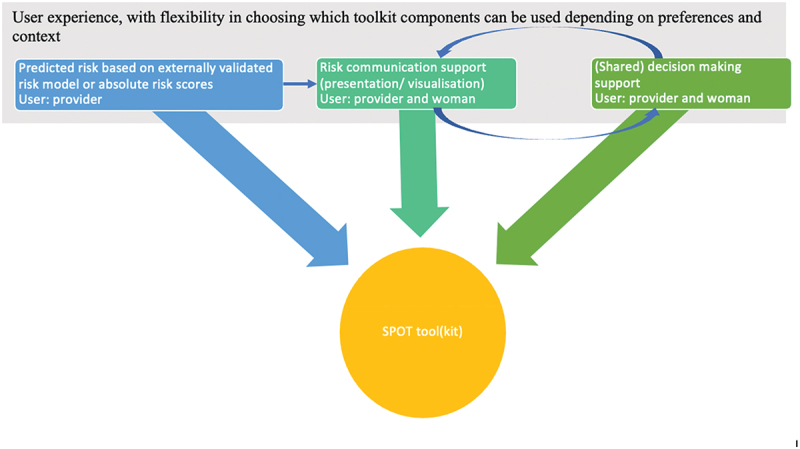
Visualization of proposed tool components.

We envision that the simple, user-friendly tool(kit) will include the following three components, as visualized in Figure 3:

1. Assessment of risk of adverse maternal and perinatal health outcomes by maternal and newborn healthcare professionals, based on a prediction model or absolute risk scores for adverse outcomes for women with certain characteristics (e.g. gestational age and type of HDP)
2. Risk communication
3. Shared decision-making between healthcare professional and pregnant women with HDP and their family members.

The tool(kit) will alert health providers regarding adverse outcomes and prompt them to discuss risks and treatment options in a manner that enhances patients’ understanding of risks and of the medical benefits of and downsides to treatment options, which are then weighed against a patient’s preferences and values. This will in turn improve quality of care, women’s care experiences and satisfaction and will possibly contribute to better health outcomes among pregnant women with HDP and their babies in the selected healthcare facilities.

## Co-creation phases

**Figure 4.**
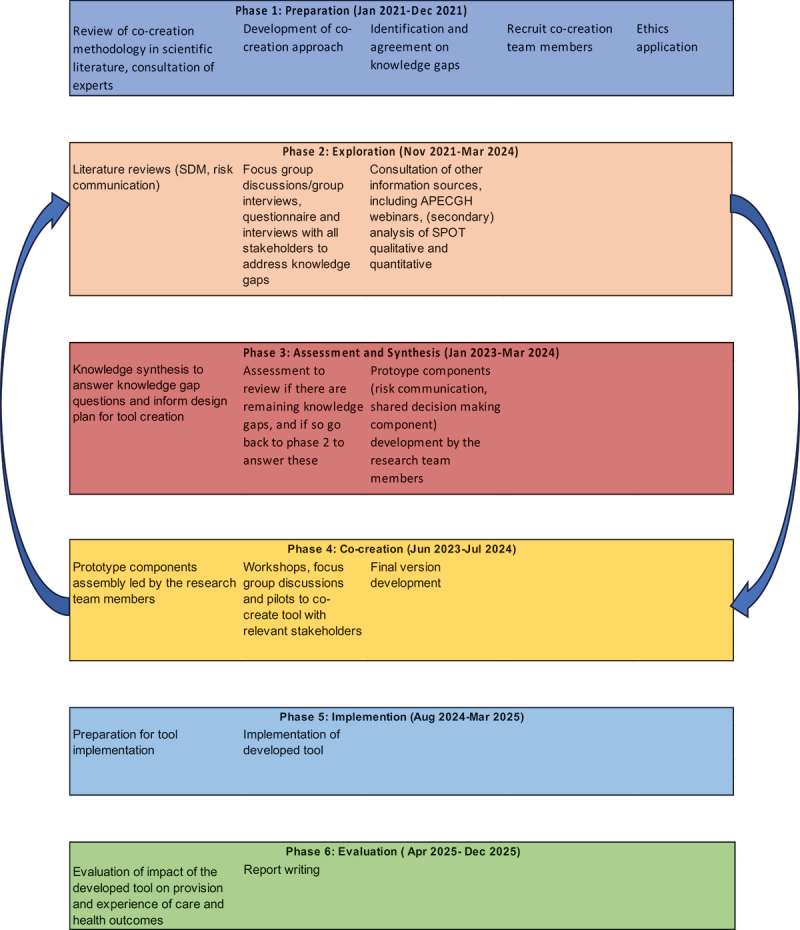
Overview of the SPOT-tool co-creation phases.

We distinguished six phases in the co-creation process: (1) preparation, (2) exploration, (3) assessment and synthesis, (4) creation, (5) implementation and (6) evaluation. Figure 4 summarizes these phases.

Phases 2–4 are iterative

The *preparatory phase* (phase 1) includes a literature review on co-creation approaches and resulted in the choice of our approach described above. In the *exploration phase* (phase 2), we use literature review, expert input, team deliberation and the aforementioned frameworks (Acceptable, Accessible, Available and promote Quality of care; Respectful Maternity Care; Justice for Global Health) to identify knowledge gaps that needed to be addressed before commencement of tool creation activities. The knowledge gaps were transformed into objectives, research questions and methods to address these (see Table 2). Additional knowledge gaps related to risks associated with HDP are addressed through epidemiological research aimed at quantifying health risks such as adverse maternal and perinatal outcomes and validating external risk prediction models for HDP in a cohort of women with HDP. Details of the epidemiological arm are published elsewhere [57].

In phase 3, the *assessment and synthesis phase (*currently ongoing), information obtained thus far will be assessed and integrated. Prototypes of SPOT tool(kit) components will be developed by the research team members.

In phase 4, the *co-creation phase*, the prototype components will be integrated into workshops, focus group discussions and pilots to co-create the SPOT tool with relevant stakeholders. Phases 2–4 will be an iterative process with the final prototype of the tool being validated, amongst others through role-play among participant groups.

In phase 5 of *implementation*, the SPOT-tool will be implemented in participating hospitals, and in phase 6, we will evaluate the tool implementation. Design of phases 3 to 6 will be described in more detail elsewhere. Below and in the rest of this article, we detail the methodology for Phase 2: Exploration, which has commenced.

## Methodology

### Study design: exploration phase

In the exploration phase, we adopted a mixed methods design, combining qualitative methods with quantitative data collection and analysis (i.e. externally validated risk models and absolute risk estimates for adverse outcomes), conducted by the study team as part of a larger study in which SPOT-Impact was embedded.

#### Study sites

Three health facilities namely Greater Accra and Eastern Regional Hospitals and the Korle Bu-Teaching hospital, in two regions in Ghana, Greater Accra, and Eastern Region, were purposively selected because of their large size, referral function, large patient volume, and required infrastructure to conduct the larger SPOT study [51], including a neonatal intensive care unit. The total annual number of deliveries in these institutions is >30,000 annually, and the estimated average incidence of HDP is about 8% or 2,400 women.

### Study participants, recruitment, and sampling

We recruited in total 120 participants, including policy makers (*N* = 3), health providers including obstetricians and midwives (*N* = 56), facility managers (*N* = 2), line managers (5), patients in stable conditions (*N* = 10), survivors of HDP (*N* = 37), male partners (*N* = 6) and a relative (1). We intended to use maximum variety sampling, a form of purposive sampling to include a wide range of participants in terms of ethnicity, age, rural versus urban domicile and socio-economic status. However, this proved too ambitious and resource intensive; it was hard to trace survivors and identify health professionals who were willing and had time to participate. Still, we could purposively sample a range of health professionals in terms of function/cadre, and a range of survivors including those with disabilities (women who are deaf and women living with Albinism), but otherwise we relied on snowball and convenience sampling. These non-probability sampling methods are appropriate for qualitative research which seeks to achieve in-depth insights regarding opinions and experiences of the population of interest, rather than achieving statistical generalizability of findings [58].

To recruit participants, we made use of the SPOT study collaborative network infrastructure including the patient-organization Action on Preeclampsia Ghana (APECGH) [59].

### Data collection methods

To address the four knowledge gaps identified in the preparation phase, we held in total 12 focus groups (FGDs; 6–10 participants) and group discussions (GDs; 3–5 participants), with clinicians (4 GDs), nurses/midwives (3 FGDs), HDP survivors (4 GDs), and a FGD with people with disabilities (hearing disabilities, blind persons) and persons living with Albinism that included two survivors and a spouse. (Focus) group discussions were homogenous (participants belonged to one category of stakeholder) to allow for more in-depth exploration amongst and comparison between the different stakeholder groups. This is important since their views on risk communication and shared decision-making may differ. Due to logistical challenges, we could organize few FGDs/GDs with survivors. Similarly, organizing FGDs with clinicians was challenging, and we reduced the number to four GDs, as well as three FGDs with midwives.

The FGDs/GDs were conducted by a trained moderator and, in most cases, with an assistant note-taker. They lasted approximately 45 min to 1 h to minimize the burden on participants. The moderator stimulated discussion by asking open-ended questions and encouraging all to contribute. We tried but failed to identify accessible venues outside facilities for FGDs/GDs with survivors but obtained access to private rooms in the health facilities that could be locked when discussions were in session. FGDs/GDs with health professionals were held inside the facility for convenience. With participants’ consent, FGDs/GDs were audio-recorded, while hand-written notes were taken as well. Topic guides are available in Annex 1.

Most FGDs/GDs and interviews were held in Akan, Ga-Dangme, and a few in English. A sign language interpreter was used for GD people with hearing disabilities. When conducted in Akan or Ga-Dangme, they were transcribed directly into English. For quality control, subsets of the transcripts will be reviewed by a second translator.

In addition, we held semi-structured individual interviews (IDIs) with patients in stable condition (*N* = 10), HDP survivors (*N* = 10), health managers (*N* = 2), policy makers (*N* = 3), clinicians (*N* = 9) and midwives (*N* = 10). In addition, we conducted four couple interviews involving an HDP survivor and her spouse. Whilst FGDs/GDs enable exploration of shared understandings, norms, and social meanings, IDIs enable more in-depth exploration of experiences and views. Moreover, an individualized approach is important given the sensitivity of especially current patients’ and survivors’ experiences of care received during an HDP episode. For health practitioners, managers, and policy makers, IDIs facilitated going beyond socially desirable responses and were easier to organize when trying to recruit busy professionals.

We interviewed one health care professional and two survivors online using the Zoom platform, and two survivors through telephone interview. As earlier studies found, the mediation by phone and internet technology did not seem to affect depth of the conversation [60,61]. It did mean that the interviews lasted longer due to internet and network connectivity issues and had to be spread out over two occasions.

We intended to incorporate the participatory method of problem tree drawing [62] in some of the FGDs to solicit stakeholders’ views on barriers to, and facilitators of, effective risk communication and shared decision-making, and how these barriers can be addressed (knowledge gaps 2 and 3). However, since we could hold few FGDs/GDs, and problem tree drawing is logistically difficult and time-consuming, we abandoned this approach.

### Document review

We intended to conduct a basic content analysis of protocols and training documents relevant to the respective facilities and medical education in Ghana to assess current guidelines, training and locally used definitions regarding risk communication, shared decision-making, and patient-centered care more broadly (knowledge gap 3). However, we were only able to identify a very limited number of existing protocols and training documents (i.e. the Ghana Patient Charter and GHS 2013 Handbook on Customer Care); an important gap in the Ghanaian medical education and health system that should be addressed.

### Observations of care interactions

To gain insight into current practices of risk communication and SDM and underpinning health care provider-client dynamics (e.g. power hierarchies) we conducted unstructured ethnographic observations on maternity wards. Observations focused on interactions between staff and clients, guided by an observation guide with prompts (see Annex 2). Ethnographic observations were paired with unstructured conversations with staff and patients as per usual practice in ethnography [63].

### Recordings of consultations & conversation analysis

After obtaining health workers’ and clients’ consent, we recorded their interactions at the out-patient department visits to conduct a fine-grained interactional analysis of patterns in risk communication and (shared) decision-making, using conversation analysis (CA) [64]. Detailed insight into health provider-client communication is important, since effective communication is one of the key elements of quality maternity care as identified by the WHO [35]. Conversation analysis will provide important new insights into how providers communicate risks or other aspects of HDP care to women with HDP, and how women respond to the providers. Furthermore, CA will enable identification of ‘conversational features’ (e.g. particular kinds of questions or their timing in the conversation) which facilitate or limit shared decision-making and patient-centered care more broadly [45].

A large body of CA studies examine provider–client interaction, exploring conversational practices such as advice giving, adherence discussions, and ‘bad news’ delivery. Shared decision-making has been studied to a limited extent only, and virtually all studies with a few exceptions focus on high-income countries [65]. Adopting this approach in a middle-income setting thus constitutes an empirical and methodological innovation and meets the call for more detailed study of abstract care and communication principles such as ‘shared decision-making’ [45,66]. Due to its innovative and resource-intensive nature (detailed transcription, translation, and analysis), we designed the CA component as a small-scale pilot.

Concretely, we obtained consent from a total of eight nurse/midwives and seven clinicians including consultants and medical officers, in two of the participating referral hospitals, to record a total of 20 consultations with normotensive women attending routine antenatal clinic and 6 consultations with women with HDP at the out-patient department. Women with HDP were selected from referral cases or identified through routine antenatal visits. We used purposive sampling, to ensure inclusion of male and female practitioners, with a range of clinical experiences with a minimum experience of 1 year. However, this was not feasible in the Eastern Region, where most of the practitioners in the maternity unit were male house officers who by default had less than 1 year of experience in maternal care provision.

A number of recordings and observations were based on our ability to identify patterns and compare across site and practitioners, whilst minimizing the burden on practitioners and keeping analysis manageable.

We had intended to provide participating health workers with a recording device which they could keep until they had recorded the desired number of consultations. However, it was more convenient for the practitioners to permit the researchers to attend the consultations and record the consultations, after they had been informed about the aim, procedure, and selection criteria, and written informed consent given.

To recruit women for the CA sub-study, the healthcare practitioners pointed out normotensive and HPD women to the researcher when they entered the midwife’s or medical doctor’s consulting rooms. In the consulting rooms, while the providers filled in their paperwork prior to care giving, the researchers informed women about the aim and procedure of the CA study and sought written informed consent. Subsequently, recording of the care consultation began.

There is a possible observer or Hawthorne effect: the act of recording may change providers’ and clients’ mode of communication. Our observations suggest that the recorder did not change how the clients interacted with clinicians but did seem to have an impact on more junior practitioners’ interaction style. They appeared somewhat uncomfortable at the start, and it is likely that some behaved with more decorum than if they were not recorded. However, earlier conversation analysis studies have shown that effects of audio and even video recording on health interactions are minimal [67,68] and that the impact of recording reduces as consultation progress [69]. Importantly, analyzing what practitioners deem best communication practice is valuable in itself. Practitioners’ ideas of best practice do not necessarily align with current policy and evidence, and CA studies have shown that health practitioners’ communicative practices do not necessarily have the intended effect. For instance, providers’ perceptions that they are giving choices regarding antenatal screening for fetal abnormalities are not necessarily matched by clients’ experience [70,71]. Other studies found that when practitioners endeavor to adopt patient-centered modes of interaction (for example, encouraging a client to set the agenda for a consultation), there are subtle, unintended ways in which choice and decision-making can be undermined [72–74]. Conversely, even where more unilateral approaches are used, and practitioners seek to impose a proposed course of action or treatment, clients can implicitly exert agency [75]. Most CA studies concern health care interactions in the UK or US (exceptions include Odebunmi, (2008) [65], Pilnick and Zayts (2012) [73]), which highlights the value of piloting a similar fine-grained interactional analysis in a middle income setting outside Europe or US.

### Co-creation workshops & APECGH webinars (knowledge gap 1–4)

We will conduct unstructured ethnographic observations during co-creation workshops and analyse APECGH webinars, to obtain insight into power dynamics and trust between different stakeholders and explore knowledge, assumptions, and preferences regarding risk communication and SDM. The observations will be open in nature, guided by research questions but not by a checklist, as explained in detail in Kielmann et [58]. Past APECGH webinars are publicly available on the internet; participants have been informed that webinars would be recorded and made available online. Participants of the webinars were informed they could turn their camera off and/or change their name.

APECGH also routinely shares ‘survivors’ stories’ through their social media channels and newsletters. The content of these will be analysed in a similar way as the webinars.

## Analysis

We analyzed the qualitative data from interviews, FGDs/GDs, and observation notes thematically [76], facilitated by Dedoose, an online qualitative data analysis software. Members of the ethnographic sub-team jointly constructed a coding tree, with codes derived deductively from the literature and inductively based on themes identified in the data. Multiple team members each coded a set of transcripts, elaborating on meanings of codes and defining more specific codes where necessary. Several transcripts were re-analyzed by another team member, followed by discussion to reach consensus regarding coding and interpretations if needed. Different team members wrote up analysis reports regarding different themes (e.g. shared decision-making; risk communication); these were then jointly discussed in team meetings, resulting in the selection of key themes and identification of relationships between themes (axial coding). The analysis of qualitative data from interviews, FGDs/GDs and observations is still in progress.

The CA of risk communication and shared decision-making analyses not just what health workers or clients say but also how they say it. The focus is on patterns in content (e.g. identified causes of death), form (e.g. pauses, word choice, grammar) and sequential placement (when statements occur). The nature and quality of communication, e.g. in what way do professionals and clients engage in ‘shared decision-making’ depend on all these features. Sequences will be selected from transcribed consultations for further analysis based on initial identification of phenomena which appear meaningful. Examples of such sequences are conversational features that are unexpected or pertaining to previously identified risk communication/SDM challenges.

## Study strengths and limitations

We need to acknowledge our study’s limitations. We were ambitious in our design but time and other resource limitations meant that we could recruit a less varied group of participants in terms of for instance disability, socio-economic classs (SEC) or educational background than we had intended. Lower SEC participants are over-represented in our study. Getting access to higher SEC patients and survivors is challenging both due to more active gatekeeping in facilities (limited access to ‘VIP’ rooms) and because higher SEC women more often decline participation. Although we conducted this study in the capital Accra and in the main city of the Eastern Region which could limit transferability of insights to facilities in rural and remote areas, many of the participating patients and survivors were from remote areas but referred to the facilities for specialized care. Furthermore, our transdisciplinary approach, use of multiple methods (IDIs, FGDs/GDs, observations, conversation analysis) and close collaboration with participating hospitals and patient organiation APECGH will deepen our insights and applicability of our results to practice.

Study findings will be detailled in forthcoming studies. In the next phases, findings will be synthesized and used to co-create a tool(kit) with all relevant stakeholders.

## Conclusion

Maternal and perinatal mortality remains a significant issue in LMICs, including Ghana. HDP accounts for a signficant and increasing proportion of maternal and perinatal mortality and morbidity. The transdisciplinary SPOT-impact study seeks to enhance the quality of care for women with HDP in terms of both clinical care and experience of care by creating a tool(kit) that can support risk prediction, risk communication, and shared-decision making. Through a series of six co-creation phases, including exploration of key knowledge gaps, we will co-create this tool(kit) with relevant stakeholders: health policy makers, health managers, clinicians and midwives, HDP patients, survivors of HDP and their partners.

In our transdisicplinary approach, we bring together medical, public health and social sciences, and policy makers, health practitioners, HDP patients and HDP survivors from the start in the study design and co-creation of the tool(kit). This both deepens and broadens our insights gained and ensures they are grounded in realities on the ground and local logics underpinning care practices [77]. By including a wide range of qualitative methods, including ethnographic observations paired with interviews and conversation analysis of recorded interactions (an approach rarely used in LMICs contexts), we can obtain detailled and in-depth insights into current practices and experiences of HDP patients and health care practitioners. This will enable a co-design that is experience- *and* practice-based and a co-creation of health care services that resonate at a cognitive and emotional level [55] and positively impact experiences of HDP patients and health care practitioners alike.

## Supplementary Material

Annex 1_Interviews and discussion topic guides.docx

Annex 2_Observation guide.docx
