# Supplementary material for: Co-creation of a toolkit to assist risk communication and clinical decision-making in severe preeclampsia: SPOT-Impact study design
Source: Glob Health Action. 2024 May 8;17(1):2336314. doi: 10.1080/16549716.2024.2336314 (PMC11080670; doi:10.1080/16549716.2024.2336314)
Supplement: Annex 1_Interviews and discussion topic guides.docx [file ZGHA_A_2336314_SM5369.docx]

## 1.1 Risk communication and SDM FGD Guide: Healthcare professionals including managers (e.g. head of maternity unit, DDNs and line/floor managers)

**Introduction**

Good morning/afternoon. My name is _______________. I am here today on behalf of the SPOT project. The SPOT study aims to improve quality of care for women with HDP. We want to do this by creating a toolkit that help in risk prediction, improve risk communication, and possibly shared decision-making between pregnant women, their family members and health professionals. We want to create this tool together with researchers, providers, facility managers, HDP survivors, their husbands and relatives. Given fact that you belong to one of the categories mentioned above role you, we would like to hear your ideas about the issues mentioned. We expect that the conversation will take about 60-90 minutes of your time.

| Domain | Topic/questions and Probe(s) |
| --- | --- |
| General introduction | **We would like to start with a general introduction about your current functions and responsibilities regarding care for pregnant women in this facility**   - Could you please describe your function and responsibilities and how long you have worked in maternity care? - Would you be so kind to share with me, two things about the care provided to pregnant women with HDP in this facility? |
| Knowledge gap 3. Assess understandings, preferences and suitable forms of a) risk communication and b) (shared) decision making | |
| Ideas about current practices on risk prediction of adverse outcomes to women with HDP and babies | 1. 1. In your view, what kind of risk(s) does a woman with HDP and her baby face? 2. 2. Can you think of and describe how maternal health professionals currently determine the risks of adverse outcomes to women with HDP and their unborn baby in their daily care delivery practices? How effective is this approach in your view? 3. 3. Does the facility or the sector have any risk prediction tool that support healthcare professionals in the delivery of care to pregnant women with HPD and their babies Why? Why not? 4. 4. What are these risk prediction tools and how do these tools look like? 5. 5. What do you like or not like about these tools? 6. 6. Do you use these tools you have mentioned. If so what makes the tool easy to use. |
| Ideas about good or bad risk communication | 1. In your view, what kind of risks do you think providers should communicate to woman with HDP when caring for them? (Probe: risks to pregnant woman, risks to baby, or also others e.g., financial 2. Who do you think should be informed about any of form risks to woman or unborn baby? Why the specific person(s)? Why not other people?   Probe: pregnant woman /husband/ spouse/relatives  5. Is that something you observe happen in practice during your routine or supervisory work? How?  6. How do you think health professionals should communicate risks to patients with HDP? Can you illustrate for me?  a. In your view, what makes this risk communication good? Why?  *7. How do* you think health professionals should NOT communicate risks to patients with HDP? Can you illustrate for me please?  a. What makes this risk communication bad? Why? |
| Understanding and preferences regarding shared-decision making (SDM)  Unintended consequences | As mentioned, we seek to create a tool, together with all stakeholders, which can enhance quality of care for women with HPD. The tool should help practitioners to assess women’s risk; it should also improve risk communication, and perhaps facilitate shared decision making between providers and clients. However, we first want to explore how all stakeholders, including you as health managers, think about shared decision making. Is it desirable, is it feasible?   1. Are you familiar with this idea of shared decision making? What does it mean to you?    1. What is your first reaction to it? 2. In your view, should women with HDP be involved in decisions regarding their care?    - - - 1. Why, please explain your answer?          2. Why not? Do you see any disadvantages of involving women in care decisions? Which ones?          3. What kinds of decisions could women be involved in during care?          4. Are there decisions they should *not* be involved in?          5. Are there specific situations in which it is not feasible or desirable to involve women in decision-making?   Probe for situations that may make shared decision making difficult e.g.   - Serious mental or physical health condition, disabilities. - Pregnant women’s socio- economic status - Any other you can think of  1. Are there others (e.g. husbands, relatives) who should to be involved in decisions about the care of women with HDP?   a. Why should these people be involved?  We would now like you to think about not involving  women with HDP in the making of decisions about their care.   - - - - 1. Might this benefit women and the unborn baby in some way?         2. Might it harm them?   Patient-centred care is usually described as taking women’ s views, preferences and needs into account in the provision of care, not just in relation to medical decision-making.   1. Do you think health profs should take the views, preferences and needs of   women with HDP into account?  a. Why or why not? |
| Knowledge gap 4. Assess personal, interpersonal and system barriers to, and facilitators of SDM. | |
| SDM in practice:  Barriers, facilitators | We’d now like to talk about practicalities. How could one actually implement SDM or patient-centred care, and what are the barriers.   1. What in your view, are barriers to SDM, what makes it hard?   Probe:  System barriers: resources incl time, guidelines, training  Interpersonal barriers: trust, power dynamics  Personal barriers: personal skills, motivations   1. a. What are facilitators of SDM, what might help providers to engage in SDM? 2. How can we address barriers to SDM? 3. How can we strengthen the facilitators to SDM? 4. And thinking more broadly about patient-centred care: what are barriers to incorporating women’s or their families’ views /preferences in care in general, not just specifically in relation to decision-making?   Probe: personal, interpersonal & system barriers.   1. How can we address these barriers and facilitate patient-centred care? |

| Knowledge gap 2. Barriers to & facilitators of meaningful co-creation | |
| --- | --- |
| Motivation for co-creation | **As mentioned, co-creation is about different stakeholders collaborating and jointly producing knowledge and health interventions. In SPOT, stakeholders include providers, facility managers, HDP of pregnancy survivors, husbands/husbands and relatives, policy makers. We’d like them to jointly co-create a tool(kit) which can enhance quality of care, based on risk prediction and principles of shared decision-making or other forms of patient-centred care.**  13. Would you be interested in participating in such a co-creation process? |
|  | 1. 14. Why: What would motivate you to participate in the process? 2. 15. Why not: What would demotivate you to participate in the process? 3. 16. What might motivate health providers to participate in co-creation? 4. 17. What would demotivate them or be a barrier for them to take part?   **Co-creation requires good collaboration between different stakeholders and having everybody’s views heard and understood and recognizing the value of survivors’ expertise in terms of experiences.**   1. 18. How can we ensure all can speak and are listened to and understood during a cocreation process?   Probe:  You could think of things like ground rules, location, or physical layout of the co-creation venue. |
| Barriers to co-creation  Power  Trust  Vulnerability-not having interests protected. |  |
| Facilitators for co-creation |  |
| Background characteristics | - Please could you tell me how long you have worked in this facility? - Kindly share with me your professional training and qualification - May I please know your age. |
| Questions, concerns recommendations | Please do you have any questions, comments or recommendations regarding care for women with HDP or our project? |

**Thank you for your time**

## A. 2.1 Risk communication and SDM interview Guide: Healthcare professionals (Clinicians, midwives/nurses).

**Introduction**

Good morning/afternoon. My name is _______________.I am here today on behalf of the SPOT project. The SPOT study aims to improve quality of care for women with HDP. We want to do this by creating toolkit that can assist risk prediction of adverse outcomes, improve and risk communication and possibly shared decision-making between pregnant women, their family members and health professionals. Given your role as a primary care provider of women with HDP in this facility, we would like to hear your ideas about the issues mentioned. We expect that the conversation will take about 45 to 60 minutes of your time.

| Domain | Topic/questions and Probe(s) |
| --- | --- |
| General introduction and ice breaker | - Could you describe your function, your responsibilities & how long you have worked in maternity care? - Could you share one thing about working with women with HDP you find satisfying and fulfilling, and one thing you find challenging? |
| Knowledge gap 3. Assess understandings, preferences and suitable forms of a) risk communication and b) (shared) decision making | |
| Ideas about good risk communication | Since one element of the SPOT tool is risk communication., we would like to hear your views on the process of communicating risks to women with HDP.   - - - 1. When caring for a woman with HDP, what kind of risks do you think providers should communicate to her? (Probe: risks to pregnant woman, risks to baby, or also others e.g. financial.       2. Who should be informed about risks? Probe: pregnant woman /husband/ spouse/relatives       3. How do you think health professionals should communicate risks? Can you illustrate for me?   1. In your view, what makes this risk communication good?  1. *How* do you think health professionals should NOT communicate risks? Can you illustrate for me please?    1. What makes this risk communication bad? |
| Understanding & preferences in SDM | The tool we seek to create should perhaps facilitate shared decision making between providers and clients. However, we first want to explore how health providers think about shared decision making. Is it desirable, is it feasible?   1. Are you familiar with this idea of shared decision making? What does SDM mean to you?   (Probe: why these reactions)  Shared Decision Making could be defined as the process of providers and clients jointly participating in health care decisions where patients’ values, preferences and circumstances (or those of family members) are taking into account.   1. In your view should women with HDP be involved in decisions about their care?    - - - 1. Why or why not?          2. Why not? |

| Unintended consequences | 1. What kinds of decisions related to HDP care could women be involved in? 2. What kind of decisions related to HDP care should they *not* be involved in? 3. Are there specific situations in which it is not feasible or desirable to involve women in decision-making? 4. Are there others (e.g., spouse/husband, relatives) who need to be involved in decisions? 5. Why should these people be involved? 6. In your view, and according to your experience, do women or others (e.g. partner, mother, in law) want to take part in decision-making re HPD treatment? 7. If so, how do they want to take part?   A broader concept than SDM is patient-centred care. This is usually described as taking women’ s views, preferences and needs into account in the provision of care, not just in relation to medical decision-making.   1. Do you think care for women with HDP should take the clients’ views, preferences and needs into account?   a. Why?  b. Why not? Any downsides to taking clients’ views preferences, needs into account? |
| --- | --- |
| Patient centered care |  |
| Knowledge gap 4. Assess personal, interpersonal and system barriers to, and facilitators of SDM | |
| SDM in practice:  Barriers, facilitators | We’d now like to focus on practicalities and talk about how one could actually implement SDM or patient centred care and what the barriers are   1. How could you *share* in decision-making with women with HDP or their husbands, relatives? What would help? 2. How could you as a provider find out about the preferences, needs and values of women who have HDP of pregnancy? 3. What are barriers to SDM, what makes it hard?   Probe:  System barriers: guidelines, training, resources incl time  Interpersonal barriers: trust, power dynamics  Personal barriers: personal Skills, motivations   1. How can we address barriers to SDM? 2. How can we strengthen facilitators of SDM? |

| Knowledge gap 2. Assess barriers to & facilitators of meaningful co-creation | |
| --- | --- |
| Motivation for co-creation | Co-creation is about different stakeholders collaborating and jointly producing knowledge and health interventions. In SPOT, stakeholders include providers, facility managers, HDP of pregnancy survivors, spouses/husband and relatives. We’d like them to jointly co-create a tool(kit) which can enhance quality of care, based on risk prediction and principles of shared decision-making or other forms of pt centred care.   1. Would you be interested in participating in such a co-creation process? 2. Why or why not?   Co-creation requires good collaboration between different stakeholders and having everybody’s views heard and understood, and recognizing the value of survivors’ expertise in terms of experiences.  spouse husband/Probe:   - How do we ensure all can speak and are listened to and understood? - Whose voices may need to be amplified? How can we achieve this?   - Consider: ground rules, location, physical layout of the venue. |

**Thank you for your time.**

## 1.1 Risk communication and SDM FGD guide: Survivors of Hypertensive Disorders of pregnancy

Introduction: The SPOT project aims to improve quality of care for women with HDP. We want to do this by producing a tool which would help to predict adverse outcomes as well as improve risk communication and shared decision-making between pregnant women’s, their family member & health professionals. Given that you are women have ever had hypertension disorders in pregnancy, we would like to have some discussions about your ideas regarding the topics just mentioned. We anticipate that the discussions may take about 45minutes to 1 hour of your time.

| Domain | Topic/questions and Probe(s) |
| --- | --- |
| 1. Assess understandings, preferences and suitable forms of a) risk communication and b) (shared) decision making | |
| Perception about when a pregnant woman is at risk and experiences of risk interactions with healthcare provider(s) | The tool we seek to develop to improve quality of care for women with HDP focuses in part on the communication of risks. We would love to hear your views on how providers should talk to women about risks.  10.When we say someone is at risk what does it mean to you?  11. What kind of risks do you think providers should inform women with HDP about when admitted in hospital for (pre)eclampsia?  Probe: risks to mother, baby, physical, mental, economic risks i.e. expenses.   1. Are there risks you think women should NOT be informed about?   We’d now like to talk about HOW doctors and nurses should talk about risks with women who suffer from hypertension/BP during pregnancy. What makes the doctors’ and nurses’ communication good in your view? E.g. certain things they should say, or is there a particular way in which they should explain certain risks?   1. How should they NOT talk about risks; what makes the drs and nurses’ communication NOT good? |
| Perception of Shared Decision Making between healthcare providers and pregnant women | We would now like to talk about shared decision making. Health professionals and researchers nowadays talk about this. It is the process of providers and clients jointly participating in health care decisions where patients’ values, preferences and circumstances (or those of family members) are taking into account   1. In your view, should women w HDP be involved in decisions re their care?    - - - 1. Why- Do you see advantages?          2. why not? Do you see any disadvantages or unintended consequences of including women’s or their families’ views and preferences about care decisions?-which ones?          3. Are there situations in which you think women w HDP should not be involved in decision-making? 2. Are there others (eg spouse, relatives) who need to be involved in decisions?    1. Why should these people be involved?    2. Are there reasons to not involve others ? 3. A broader concept than shared decision-making is patient-centred care. This is usually described as taking women’ s views, preferences and needs into account in the provision of care, not just in relation to medical decision-making. Do you think doctors and nurses should take the views, preferences and needs of women with HDP into account? Why? /Why not? |
| Knowledge gap 4. Assess personal, interpersonal and system barriers & facilitators to SDM. | |
| Barriers to & facilitators of SDM/patient centred care | 1. Are there situations in which it is not feasible or desirable for women to be involved in decision-making re their care? 2. What are barriers to SDM, what makes it hard? 3. What would enable providers and clients to make decisions together? 4. And what would help providers to find out about women’s needs, values and preferences more generally? How can they take these into account in care? |
| Barriers to and facilitators for Co-creation | As mentioned we seek to co-create a tool to improve quality of care for women with HDP. Co-creation requires different stakeholders collaborating and jointly producing knowledge and health interventions. In SPOT, stakeholders include providers, facility managers, health professionals, HDP of pregnancy survivors, spouses and relatives.   1. Would you be interested to help co-create a tool to improve QoC focused on risk prediction, risk communication and shared decision making? Why/why not? 2. What would motivate you? 3. What would demotivate you or what are barriers to your participation? 4. What might make it hard for you **as survivors** to work together with providers, facility managers, health professionals, HDP of pregnancy survivors, spouses and relative?   Probe:   - Do you think hierarchies or power differences may affect co-creation between women, male partners, relatives, health profs? - Trust may matter for instance trust between you and HPs. Do you trust HPs? Why/why not? Do you think HPs trust you? - Having everybody’s views heard and understood, recognizing the value of patients’ or survivors’ expertise in terms of experiences. Do you foresee any challenges here?  1. What might make It easier to collaborate for you?   (probe: Think for instance about how different stakeholders should act and relate to each other; location, or physical layout of the co-creation venue. |
| Questions, concerns recommendations | Please do you have any questions, comments and recommendation for care for women with HDP or for our co-creation project? |

***Thank you for your time***

## B. 2.1. Risk communication and SDM Interview Guide: Pregnant women with Hypertensive Disorders in Pregnancy (and are in a stable condition)

**Introduction**

Good morning/afternoon. My name is _______________ and I am from ___________________.

I am here today on behalf of the SPOT project. The project aims to improve quality of care for women with HDP. We want to do this by with researchers, providers, facility managers, HDP survivors, their husbands and relatives to create a tool that will help health foretell pregnancies that may have more problems later and possibly shared decision-making between pregnant women, their family members and health professionals to improve the quality of care (QoC). Given that you have a high blood pressure and are receiving care, we’d like to invite you to take part in an interview, to hear your views about the way in which providers talk to you about your health problem and make decisions. The interview/conversation will take about 30 minutes of your time.

| **Domain** | **Topic/questions and Probe(s)** |
| --- | --- |
| General introduction and ice breaker | Kindly share with me how you and the baby are feeling today?  What food have you being craving for or do not crave for today? |
| **Knowledge gap 3. Assess understandings, preferences and suitable forms of a) risk communication and b) (shared) decision making** | |
| **Preferences of pregnant women with HDP regarding (risk) communication** | The doctors found that there is an issue with your BP and you are now in the hospital for it. The doctors and nurses will be talking to you about all sorts of things.   1. Is there anything you like about the way in which they talk to you? 2. What do you NOT like so much about the way in which they talk to you? 3. Do you feel you understand the things the doctors and nurses are telling you about your pregnancy? Are there things you do not understand? 4. Do you feel they talk to you kindly? With respect? Why/why not? 5. Do you feel you can ask them questions? Why/why not? 6. Do you feel they listen to you? 7. What things would you like to know about in relation to your condition of HDP/BP?   Probe: potential treatment, treatment side-effects, consequences of HDP for your baby, for yourself a. Have these things been discussed with you?   - 1. Do you feel you received enough information about this?   2. Were you able to ask all your questions  1. How would you have liked to be told about your condition?   Probe e.g. at particular time or moment / by particular practitioner?   1. Is there somebody else you think doctors, midwives or nurses should also talk to about your condition and treatment? Who? E.g. spouse/husband, relative? 2. Is there anything you would *not* like to know about? |
| **Appropriateness of the term & concept SDM** | We would now like to talk about shared decision making. Health professionals and researchers nowadays talk about this. It is the process of providers and clients jointly making health care decisions. Doctors and nurses take patients’ values, preferences and circumstances (or those of family members) into account when taking decisions about women’s care or treatment.   1. Would you like your dr/midwife to involve you in decisions? Why/why not? 2. How do you think they could involve you? 3. Are there others (eg spouse/husband, relatives) who need to be involved in decisions?    1. Why should these people be involved? 4. Are there people you think should NOT be involved in decisions?   a. Why?   1. More generally, do you think providers should take women’s needs and preferences into account when caring for them? Why/why not? |
| Background characteristics | Please could you tell me your religious affiliation?  Do you have a spouse/husband?  May I please know your age?  Kindly share with me your occupation and your education?  And your spouse, may you share his occupation and education?  *(The questions that follow should only be asked if this information cannot be obtained from the medical records of the participant)*  How many children do you have? Have you ever had miscarriage? a stillbirths? or a baby that died very soon, within 4 weeks after birth? If yes to any of them how many? How months in or after pregnancy did this occur? Have you had a HDP in a previous pregnancy apart from the current experience? |

| Questions, concerns recommendations | Please do you have any questions, comments or recommendations regarding care for women with a condition like yours, or for our study? |
| --- | --- |

***Thank you for your time.***

## C. 1.1 Risk communication and SDM Interview guide: Spouses /relatives of women who have had HDP

| Domain | Topic/questions and Probe(s) | |
| --- | --- | --- |
| *Background/Introduction* | - How are you related to a woman who has or has had HDP? - Would you mind telling me a bit about your spouses/husband’/relatives HDP experience(s)? | |
| Knowledge Gap 3. Assess understandings, preferences and suitable forms of a) risk communication and b) (shared) decision making | | |
| Experiences & Perceptions & preferences of risk communication | | The tool we seek to develop to improve quality of care for women with HDP should help providers assess women’s health risks and help them communicate risks to women. We’d love to hear your views about HOW health providers should talk about risks with women who suffer from hypertension/BP during pregnancy and their relatives.   - - - 1. When we say someone is at risk what does it mean to you?       2. Risk is really about the chance that something bad happens to you or your baby. Do you remember what, if anything, your spouse/relative was told about any risks during the time she was admitted?       3. How did you feel about how they talked to your spouse or yourself about risks?   What did you like?  What did you NOT like?  Did you feel they were clear? What was not clear, can you give examples?  Did you feel they talk to you kindly? With respect? Why/why not?  Did you feel you could ask them questions? Why/why not?  What kind of risks do you think providers should inform women with HDP about when admitted in hospital for (pre)eclampsia? (probe: risks to mother, baby, physical, mental, economic risks ie financial implications)   - - - 1. Are there risks you think women should NOT be informed about?       2. If you were to advice doctors and nurses about how to speak to their patients about risks - what would you say to them? What makes their communication good?   You could think E.g. about how they explain risks, or at what kind of moments they talk to patients.   1. What should doctors and nurses AVOID when they talk to HDP patients about risks?; what makes the doctors communication NOT good? |
| Perception of Shared Decision Making | | We would now like to talk about shared decision making. Health professionals and researchers nowadays talk about this. It is the process of providers and clients jointly participating in health care decisions where patients’ values, preferences and circumstances (or those of family members) are taking into account   1. Did the Dr or midwife involve you, or your spouse/relative in decisions of the care of your relative who had BP in pregnancy?    - - - 1. If yes: How? What kind of decisions? *(If the response to question 8 is no, then skip to question 10).* 2. Would you have liked to be involved or would you have liked your spouse/relative to be involved? Why? 3. In general, when doctors make decisions about women’s treatment and care, do you feel they should involve others?    1. Who?    2. Why/why not?    3. In what ways? |
| Knowledge gap 4. Assess personal, interpersonal and system barriers & facilitators to SDM. | | |
| Barriers to & facilitators for SDM and patient-centred care | | We’d now like to focus on practicalities and talk about how one’s provider could actually involve clients in decisions, and what the barriers are.   1. What in you view are barriers to SDM: what makes it hard for providers and clients to jointly make health care decisions?   Probe: barriers affecting health professionals vs women  System barriers: guidelines, training, resources incl time  Interpersonal barriers: trust, power dynamics  Personal barriers: personal Skills, motivations   1. What are facilitators of SDM, what would make it easier for women to be involved in decisions regarding their care? 2. Providers could also more generally take women’s views or preferences into account when providing care, not just specifically in relation to decision-making. (This is also called patient centred-care). What would help them to take account of women’s views and preferences?   Probe – System: guidelines, training, resources incl time  Interpersonal: trust, power dynamics  Personal: personal Skills, motivations   1. What would make it harder for providers to take women’s views and preferences into account? |
| Barriers to and facilitators for Co-creation | | As mentioned we seek to co-create a tool to improve quality of care for women with HDP. Co-creation means that different stakeholders work together. In SPOT, stakeholders include providers, facility managers, health professionals, HDP of pregnancy survivors, spouses and relatives.   1. Would you be interested to help co-create a tool to improve QoC focused on risk prediction, risk communication and shared decision making? Why/why not? 2. What would motivate you? 3. What would demotivate you or what are barriers to your participation? 4. What might help you **as spouse or relative of survivors** to work together with providers, facility managers, health professionals, HDP of pregnancy survivors, other spouses and relative?   Probe:   - How can we ensure that everybody’s views are heard and understood? How to ensure all can speak and are listened to and understood? - Whose voices especially may need to be amplified? How can we achieve this? - Consider e.g. ground rules, location, physical layout of the venue.) |
| Background characteristics | | Please could you tell me your:   - Religious affiliation. - Highest level of education & occupation. - How many children do you have? |
| Questions, concerns recommendations | | Please do you have any questions, recommendation for care for women with HDP or for our co-creation? |
