# Supplementary material for: Co-creation of a toolkit to assist risk communication and clinical decision-making in severe preeclampsia: SPOT-Impact study design
Source: Glob Health Action. 2024 May 8;17(1):2336314. doi: 10.1080/16549716.2024.2336314 (PMC11080670; doi:10.1080/16549716.2024.2336314)
Supplement: Annex 2_Observation guide.docx [file ZGHA_A_2336314_SM5368.docx]

## E.1.1 Observation guide

**For observations of care the maternity ward or at the OPD (conversation analysis sub-study)**

| **Observation Guide** | |
| --- | --- |
| **Domain** | **Observation Prompts regarding interactions between with provider(s) and clients/relatives on the maternity ward (objective 4) or at the OPD (conversation analysis sub-study, objective 2)**  Note: Prompts below serve to direct attention and are suggestions; do NOT treat as ‘check box’. Focus on creating rich in-depth descriptions of provider-client interactions and care practices. |
| **Description of context** | - Brief description of the state/condition of the pregnant woman at arrival in the location where observation is being carried out.   - Who accompanied the pregnant woman?   - What location /department is the pregnant woman with HDP first received?   - What cadre(s) of health professionals are involved in the woman’s care? Who else is present apart from the healthcare providers? Why are they there? What are they doing? |
| **Interactions between provider-client:**  **Risk communication** | Describe:   - Any verbal or non-verbal interactions between pregnant woman/relatives and provider - How did the interaction about HDP begin? - What issues were discussed? - Was a relative or spouse of the pregnant woman present during the interaction? - Was any information about risk shared with the pregnant woman/ spouse/relatives/family members? - In what manner did the interaction/conversation go? - Any differences in interaction between the different categories of health profs and the pregnant woman/spouse/ relatives? Did different actors play a different role? In what sense? - Any questions asked or concerns raised by pregnant woman/spouse/ relatives/family members during the interaction with health professional? Which questions or concerns?   Any clues from observations or conversations about why the interaction went as described?   - Describe apparent socio-economic, cultural or power related factors that may have influenced risk communication - Describe various perceived contextual and organizational related factors that influences risk communication - Describe other observed factors influencing care providers’ communications with pregnant woman and relatives |
| **Description of shared-decision making process** | Describe :   - How the interaction is carried out between provider, client and relatives (e.g. verbal & non-verbal behaviours, client’s & relatives’/spouses’ expressions, reactions during care decision-making interaction; use of material objects). - Include description of key issues healthcare providers, pregnant woman and any relatives discussed about the management of their HDP condition - Any preferences pregnant woman and any relatives mentioned during the care management interactions - How healthcare providers responded to the pregnant woman and any relatives during the interactions? - Describe any interactions/conversations amongst healthcare providers themselves after the consultation with pregnant woman and relatives concerning the client’s care. - Describe apparent socio-economic, cultural, organizational, or power related factors that influence the interactions between providers, clients and relatives, and the decision-making process? |
